# Radiosensitization Effect of Talazoparib, a Parp Inhibitor, on Glioblastoma Stem Cells Exposed to Low and High Linear Energy Transfer Radiation

**DOI:** 10.1038/s41598-018-22022-4

**Published:** 2018-02-26

**Authors:** Paul Lesueur, François Chevalier, Elias A. El-Habr, Marie-Pierre Junier, Hervé Chneiweiss, Laurent Castera, Etienne Müller, Dinu Stefan, Yannick Saintigny

**Affiliations:** 1 LARIA, iRCM, François Jacob Institute, DRF-CEA, Caen, France; 2 UMR6252 CIMAP, CEA - CNRS - ENSICAEN - Université de Caen Normandie, Caen, France; 3 Radiotherapy Department, Centre François Baclesse, Caen, France; 4 CNRS UMR8246, Inserm U1130, UPMC, Neuroscience Seine-IBPS, Sorbonne Universities, 75005 Paris, France; 5 Plateforme de sequencage haut debit, Centre François Baclesse, Caen, France

## Abstract

Despite continuous improvements in treatment of glioblastoma, tumor recurrence and therapy resistance still occur in a high proportion of patients. One underlying reason for this radioresistance might be the presence of glioblastoma cancer stem cells (GSCs), which feature high DNA repair capability. PARP protein plays an important cellular role by detecting the presence of damaged DNA and then activating signaling pathways that promote appropriate cellular responses. Thus, PARP inhibitors (PARPi) have recently emerged as potential radiosensitizing agents. In this study, we investigated the preclinical efficacy of talazoparib, a new PARPi, in association with low and high linear energy transfer (LET) irradiation in two GSC cell lines. Reduction of GSC fraction, impact on cell proliferation, and cell cycle arrest were evaluated for each condition. All combinations were compared with a reference schedule: photonic irradiation combined with temozolomide. The use of PARPi combined with photon beam and even more carbon beam irradiation drastically reduced the GSC frequency of GBM cell lines *in vitro*. Furthermore, talazoparib combined with irradiation induced a marked and prolonged G2/M block, and decreased proliferation. These results show that talazoparib is a new candidate that effects radiosensitization in radioresistant GSCs, and its combination with high LET irradiation, is promising.

## Introduction

Glioblastoma (grade IV, WHO classification) is the most common and most deadly glioma subtype in adults, occurring at a rate of 0.59 to 3.69 per 100,000 persons^1^. The standard of care consists of extensive neuronavigation-guided neurosurgery, followed by radio-chemotherapy 4 weeks later. 60-Gy (photons) conformational or intensity-modulated radiotherapy is usually delivered with concomitant temozolomide^2^. Monthly adjuvant cyclic temozolomide has to be continued until progression or for 1 year.

Despite this multimodality treatment, the median overall survival is limited to 14.6 months^2^. Thus, nearly 100% of patients relapse, and over 75% of relapses are “in-field” recurrences, meaning they are located inside of the 95% isodose line^3^. As a consequence, glioblastoma (GBM) is considered a chemo- and radioresistant tumor.

Glioblastoma stem cells (GSCs) are a specific subpopulation of GBM cells with properties of tumor stem cells, such as unlimited proficiency, self-renewal, differentiation, and metastatic abilities; they also have high DNA repair capactiy^4^, which explains a major part of the radioresistance of GBM. Indeed, DNA damage response factors, such as ATM serine/threonine kinase (*ATM*), ATR serine/threonine kinase (*ATR*), poly(ADP-ribose) polymerase 1 (*PARP1*), and checkpoint kinase 1 (*CHK1*), are upregulated in GSCs^4,5^. Furthermore, GSCs exhibit drug resistance, including that to temozolomide. Thus, a widely accepted hypothesis for explaining the poor patient survival posits that the GSC population responds differently to temozolomide or radiation therapy than the nonstem cell population.

PARP1 has an important cellular function, detecting the presence of damaged DNA and then activating signaling pathways that promote appropriate cellular responses. PARP1 is involved in base excision repair (BER), allowing the recruitment and activation of BER factors, and consequently facilitating the repair of DNA single-strand breaks (SSBs)^6^. PARP1, 2, and 3 are equally involved in other cellular mechanisms, such as chromatin remodeling and DNA double-strand break repair^7^.

PARP inhibitors (PARPis) first entered clinical trials in 2003 in combination with the monomethylating agent temozolomide for patients with advanced solid tumors^8,9^. Their development has been accelerated since the concept of synthetic lethality appeared in homologous recombination-deficient cells that were exposed to PARPis^10^. PARPis have been considered as potential radiosensitizers due to increasing the number of unrepaired DNA DSBs. Dungey *et al*. showed that the radiosensitizing effect of PARPis is S-phase dependent^11^. This replication-dependent mechanism allows adjacent healthy tissues to be protected while sensitizing highly proliferative tumors. Nine preclinical studies and one phase I study have investigated PARPis as radiosensitizers for glioma cells^9^. Enhancement ratios ranged between 1.08 and 1.93 in *in vitro* studies^9^, and the only published phase I study assessed the safety of the combination PARPi (veliparib) and irradiation^12^.

Heavy ion beams have distinct physical and biological characteristics, contributing to the overall improved risk-benefit profile in radiation therapy (RT). Due to low dose deposition inside of the entry channel of the beam and high local dose deposition in the so-called Bragg Peak^13^, dose conformality can be improved, and low- and medium-dose regions in normal tissue surrounding the target volume can be reduced. Carbon ions also have higher relative biological effectiveness (RBE), which has been shown to be between 2 and 5 in GBM cell lines^14^. The use of a heavy ion beam could be another approach to bypassing GSC radioresistance^15^. Carbon ion irradiation targets otherwise untreatable hypoxic and radioresistant diseases, such as GBM^15^.

In this study, we evaluated whether the new PARP inhibitor talazoparib^16^ could be used as a radiosensitizer for radioresistant GSC in association with conventional low linear energy transfer irradiation (LET) and with high LET particle therapy, such as a carbon ion beam.

## Material and Methods

### Cell lines

Two GSC cell lines, R633 and TG1, were obtained from Dr. H. Chneiweiss (UPMC, Paris). These GSCs were isolated from neurosurgical biopsy samples of human GBMs, and their stem-like and tumor-initiating properties have been previously reported^17–20^.

### Cell line characterization by sequencing

The exons of 69 genes that have been implicated in DNA repair and specifically in homologous recombination were sequenced for each cell line. Dry pellets of 1–2 million cells were frozen and used for sequencing. Considering that the best responders to chemotherapeutic agents present with genomic alterations in homologous recombination genes and consequently have the best overall survival, this gene panel (supplementary table S1) was based on the tumor genomic profiles of long-term survivors of ovarian cancer (data from The Cancer Genome Atlas [TCGA] database). Reads were sequenced 2 × 75 bp paired-end on an Ilumina NextSeq. Bioinformatics analysis was performed with 5 variant callers^21–23^ (Supplementary Figure S1).

### Drug preparation

All drugs were used at concentrations as close as possible to clinically used concentrations. Temozolomide (SigmaAldrich®, St. Louis, MO, USA) was used at a concentration of 10 μM, corresponding to CSL or brain tissue concentrations of 75 mg/m^2^ after oral administration as prescribed in clinical practice^24–26^. Talazoparib (Bertin Pharma®, Paris, France) was prepared at 0.05 μM in accordance with maximum plasma concentration obtained from administration of 1 mg in human patients^27^. Talazoparib was compared with 2 other PARPis—olaparib (Tebu-bio®, Le Perray-en-Yvelines, France) and AG 14361(Tebu-bio®)—at respective concentrations of 5 μM and 2 μM^28^. These concentrations were tested separately and did not induce any significant cell mortality according to trypan blue assays. All drugs were prepared in DMSO, were added to the medium 2 hours before irradiation, and were left in the cell culture medium until the end of the experiment. Negative control samples were treated with the highest DMSO concentration used for the test samples without exceeding 0.2%.

### Irradiation

For X-ray exposures as performed in canonical irradiation, doses between 1 and 8 Gy were used. The photon beam was delivered with an energy of 225 kV and an intensity of 9.5 mA, corresponding to a dose rate of 2 Gy/min on the XradSmart 225cX irradiator (Precision X-Ray®, North Branford, CT). For accelerated carbon ion exposure as used in hadrontherapy protocols, the dose reference was 2 Gy, assuming an RBE of 2. Doses reported are absorbed doses (physical doses) according to International Atomic Energy Agency recommendations^29^ and the Heavy Ion Medical Accelerator in Chiba visiting committee^30^. Irradiations were realized on laminin-coated flasks on the IRRABAT beam line from Grand Accélérateur National d’Ions Lourds (Caen, France)^31^ and the CATANA beam line from the Istituto Nazionale di Fisica Nucleare - Laboratori Nazionali del Sud (Catania, Italy)^31^. On each beam line, carbon ions (^12^C) were used with a linear energy transfer (LET) evaluated at 50 keV μm^−1^ to the cells^31,32^.

### Cell cycle analysis

Cell cycle analysis was performed with FX Cycle violet stain (Invitrogen, Carlsbad, CA, F10347), and S phase analysis was conducted with EdU labeling (Click-iT Plus EdU Alexa Fluor 647 Flow Cytometry Kit®, Thermo Fisher Scientific, Waltham, MA, USA). Samples were treated on a Gallios flow cytometry system (Beckman Coulter, Brea, CA, USA)^33^. Data were analyzed with FCS Express 6 + software (DeNovo Software®, Glendale, CA, USA).

### Proliferation assay

Cell proliferation was evaluated with a CellTrace® Far Red kit (Thermo Fisher Scientific®, Waltham, MA, USA). This labeling dye allows one to monitor cell divisions^34^. The Celltrace Far Red stain crosses the plasma membrane and covalently binds to all free amines on the surface and inside of cells. The stable, well-retained fluorescent dye provides a consistent signal, even after several days in a cell culture environment. Thus, the fluorescent dye signal is inversely related to the number of cell divisions. Cells were stained before irradiation, and proliferation was measured 5 days after 4-Gy photonic irradiation. Data were analyzed with FCS Express 6 + software (DeNovo Software®, Glendale, CA, USA).

### Determination of GSC frequency

The GSC subpopulation (%) was evaluated in 96-well plates, seeded at a cell density of 1, 3, 6, 12, 19, 25, 37, 50, 75, 100, 150, or 200 cells/well/200 μl. The number of wells containing at least one primary sphere was evaluated after 12 days of culture, and the percentage of positive wells was determined according to a Poisson distribution. The analysis of the frequency of sphere-forming cells, a surrogate property of brain cancer stem-like cells^35^, was performed with ELDA software, available at http://bioinf.wehi.edu.au/software/elda/, as previously described^36^.

### Statistics

Statistics and figures were edited with GraphPad Prism® 7.0 (GraphPad Software Inc., La Jolla, CA, USA), Excel 2010 (Microsoft®, Redmond, WA, USA), XLSTAT 2016® (Addinsoft, New York, NY, USA), and ELDA^36^ software for extreme dilution limit assays (Supplementary data file S1). The level of significance was set at *p* < 0.05. Error bars correspond to the 95% confidence interval. Statistical analysis of proliferation was performed by nonparametric Kruskall-Wallis t-test.

## Results

### Genetic characterization of TG1 and R633 cell lines

The exons of 69 genes implicated in homologous recombination were sequenced for each cell line in order to identify deleterious mutations of genes involved in DNA repair, the defects of which could lead to enhanced anticancer effects or synthetic lethality when cells are exposed to PARPis and irradiation. After applying our pipeline (Supplementary Figure S1), the results were filtered according to sequencing depth and quality, allele frequency, variant frequency in Euro-American and Afro-American populations, and the predicted deleterious characteristics of the mutations.

Few genetic profiles were predictive of increased sensitivity to irradiation or to PARPi. We confirmed that the phosphatase and tensin homolog (*PTEN*) gene was deleted in the R633 cell line, as described, with no expression by western blot analysis^18^ (Table 1). An additional deleterious heterozygous missense mutation was detected in the SWI/SNF-related, matrix-associated, actin-dependent regulator of chromatin, subfamily a, member 4 (*SMARCA4*) gene for the same cell line. *SMARCA4* is known to be involved in chromatin remodeling and plays a substantial role in the homologous recombination repair pathway^37^. Then, a splice site mutation was detected on BRCA1-Interacting Protein 1 (BRIP1) gene. BRIP1 acts late in the Fanconi anemia pathway, after FANCD2 ubiquitination. involved in the repair of DNA double-strand breaks by homologous recombination in a manner that depends on its association with BRCA1. For the TG1 cell line, a deleterious missense substitution was found in *ATM*, which could alter signaling related to DNA double-strand breaks. In addition, R633 cells are known to have mutated tumor protein 53 (*P53*)^18^, in contrast to wild-type TG1 cells. Both cell lines were IDH1-2 wild type^18^. Thus, the 2 cell lines had different genetic profiles, which could lead disparate responses to PARPi or irradiation.

**Table 1:** Exome analysis results for the 69 genes.

Cell line	Gene	Event	Mutation
TG1	ATM	Missense substitution	p.Ser1383Leu
R633	BRIP1	Subsitution with impact on splicing	/
R633	PTEN	Deletion	PTEN (−/−)
R633	SMARCA4	Missense substitution	p.Tyr475His

### Induction of a prolonged G2/M block

The 2 cell lines were exposed to vehicle, talazoparib, temozolomide, or talazoparib + temozolomide with or without irradiation (4 Gy, X-rays). Cells were fixed with ethanol at 24, 48, 72, and 96 hours after irradiation, and cell cycle analysis was performed at these time points.

**Figure 1:**
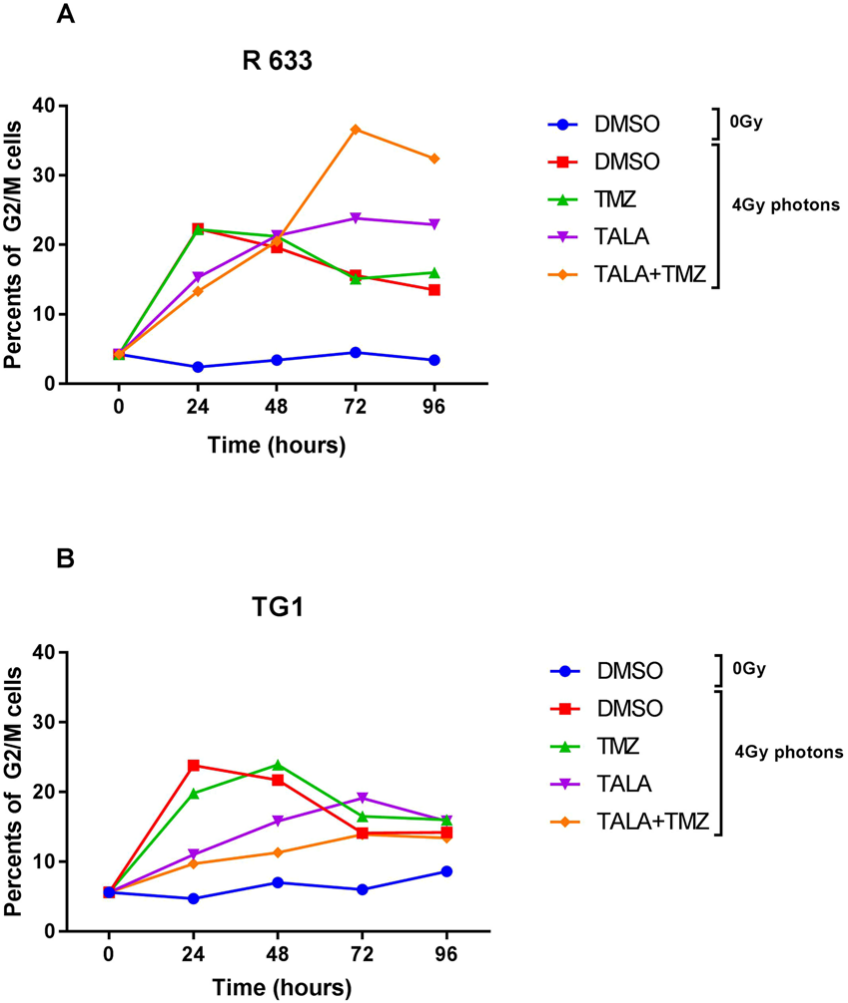
Impact on G2/M block of 4 Gy photonic irradiation combined with different radiosensitizers for (**A**) R633 and (**B**) TG1 cell lines.

For the R633 cell line, no G2/M block was observed for non irradiated vehicle-treated cells (Fig. 1A: Control DMSO). The addition of temozolomide, talazoparib, or both, without any irradiation, induced G2/M block but did not impact S phase (Supplementary Figure S2). In contrast with control samples, a sub-G1 population appeared after exposure to temozolomide, talazoparib, and especially talazoparib + temozolomide from 48 hours to 96 hours post irradiation.

A single dose of 4 Gy to vehicle-treated cells induced rapid G2/M block, peaking at 24 hours after irradiation. Afterwards, the cell cycle restarted, and the G2/M cell fraction decreased (Fig. 1A: DSMO + 4 Gy). Treatment with temozolomide and photonic irradiation caused no further increase in the G2/M block (22% of cells blocked in G2/M), and no major difference could be observed between temozolomide + 4 Gy and DMSO + 4 Gy. When talazoparib and, particularly, talazoparib plus temozolomide were combined with irradiation, the increase in the G2/M-blocked cell fraction was slower (Fig. 1A: talazoparib + 4 Gy; talazoparib + temozolomide + 4 Gy). However, the peak amplitude was higher, reaching 36.6% at 72 h for talazoparib + temozolomide + 4 Gy, and the presence of PARPi seemed to prevent the cell cycle from restarting. The fraction of cells that were blocked in G2/M stayed between 23% and 32% through 96 hours after irradiation for the talazoparib + 4 Gy and talazoparib + temozolomide + 4 Gy samples, respectively. In all irradiated samples, a sub-G1 population was observed (Supplementary Figure S3).

In the TG1 cell line, the addition of temozolomide and, above all, talazoparib or talazoparib + temozolomide, without any irradiation, strongly impacted S phase (Supplementary Figure S4); only 4% of the whole TG1 population was in S phase 96 hours after exposure to temozolomide and talazoparib versus 17% for the control sample. The results of the cell cycle analysis of irradiated cells (Fig. 1B: DMSO + 4 Gy) and irradiated cells exposed to temozolomide (Fig. 1B: temozolomide + 4 Gy) were similar to those of the R633 cell line. The addition of temozolomide did not change the proportion of cells in G2/M phase compared with controls. The addition of talazoparib alone or with temozolomide, combined with irradiation, led to a later and lower G2/M block than in controls (Fig. 1B: talazoparib + 4 Gy; talazoparib + temozolomide + 4 Gy); at 96 hours, all levels of G2/M block were equivalent to those induced by irradiation alone. The analysis of EdU incorporation revealed an antiproliferative effect in the presence of talazoparib from the Day 3 postirradiation, with a significant decrease in the proportion of cells in S phase (6.1% to 7.4%) as a function of talazoparib combination, compared with 18% after single irradiation combined with temozolomide (Supplementary Figure S5).

In both tested cell lines, the combination of talazoparib with irradiation effected a more prolonged accumulation of cells in G2/M phase. This finding was more significant for the R633 versus TG1 cell line. For the TG1 cell line, the combination predominantly impacted S phase (Supplementary Figures S3 & S5), which was delayed for a fraction of cells and arrested for others. Consequently, fewer cells were able to progress to the next phase, thus inducing a reduction in the accumulation of cells in G2/M phase.

### Combinations of PARPi plus irradiation decrease cell proliferation

Long-term GBM survivors are more likely to have lower proliferation rates compared with typical GBM survivors^38^. Consequently, the proliferation index could be considered a prognostic factor. Therapies that are able to reduce it and slow disease progression could be valuable for clinical practice. Here, the proliferation index was determined from the number of cell generations during the 5 days following exposure to the various therapeutic combinations. Cells were seeded in 6-well plates at a concentration of 300,000 cells per well.

**Figure 2:**
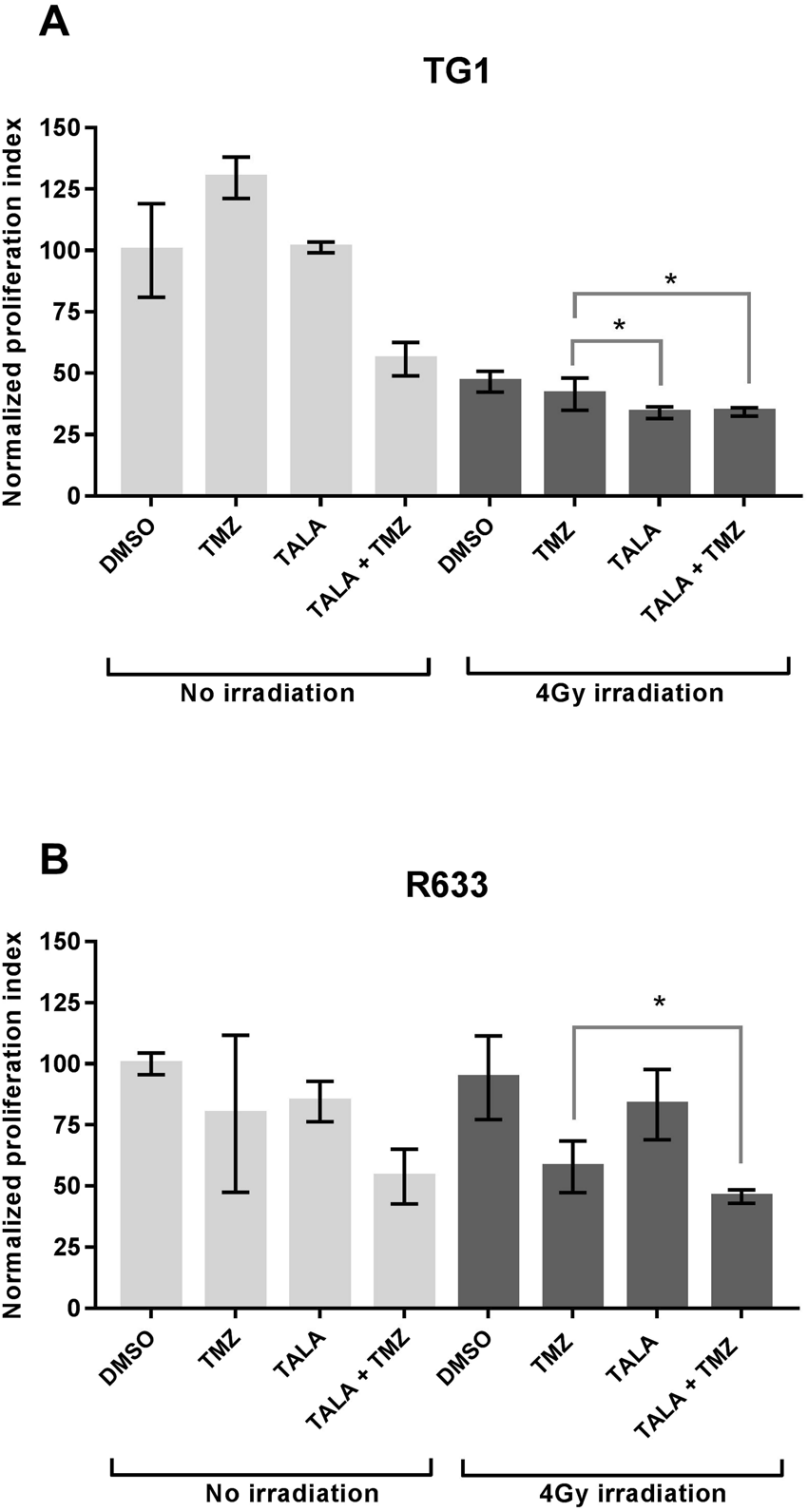
Proliferation indexes after exposure photonic irradiation (4 Gy) combined with talazoparib (TALA) +/− temozolomide (TMZ) (**A**: R633 cell line, **B**: TG1 cell line). Combinations with significant decrease in GSC fraction (*p* < 0.05) in comparison with temozolomide + 4 Gy are marked with*. Nonsignificant differences are marked with ns.

For the R633 cell line, without any irradiation, only the combination of temozolomide and talazoparib reduced the proliferation index significantly, by 46% (*p* = 0.004), in comparison with the control sample (Fig. 2A). A single 4-Gy dose of photonic irradiation alone did not impact cell proliferation (*p* = 0.74), but when irradiation was combined with temozolomide, the proliferation index was reduced by 38%. In comparison with photonic irradiation combined with temozolomide (Stupp combination), the addition of talazoparib without temozolomide did not provide any benefit in terms of proliferation. None of the other combinations that included talazoparib decreased the proliferation index significantly more than the Stupp combination.

For the TG1 cell line, the impact of the combinations on proliferation was clear. Without any irradiation, talazoparib with temozolomide decreased the proliferation index by 44% (*p* = 0.04) when compared with the DMSO control (Fig. 2B). Irradiation alone (DMSO control) reduced the proliferation index by 54% (*p* = 0.002). For talazoparib + irradiation and talazoparib + temozolomide + irradiation, the proliferation indexes were equal to 3.27 and 3.30, respectively (Fig. 2B). These results differed significantly from those obtained for the Stupp combination (*p* < 0.04), meaning that the addition of talazoparib as a radiosensitizer had a higher antiproliferative effect than the Stupp combination.

These results were consistent with the decrease in the proportion of cells in S phase observed in the EdU cell cycle analysis.

### Determination of GSC fractions for TG1 and R633 cell lines

GSCs are functionally defined by the ability of a single cell to form tumorspheres *in vitro*. According to the commonly observed heterogeneity within a tumor, only a fraction of cultured cells correspond to GSCs. Determining the spontaneous GSC fractions for TG1 and R633 cell lines is essential for the subsequent evaluation of the impact of PARPis and irradiation on GSCs. The impact of a specific treatment on the tumorsphere-forming potential of GSCs can be evaluated by limiting dilution assay, which allows an estimation of stem-like cell frequency.

Nonirradiated cells were dissociated and seeded at different densities in a nonadherent 96-well plate (cf. Materials and Methods). After 12 days of culture, the number of positive wells containing at least 1 neurosphere was counted.

**Figure 3:**
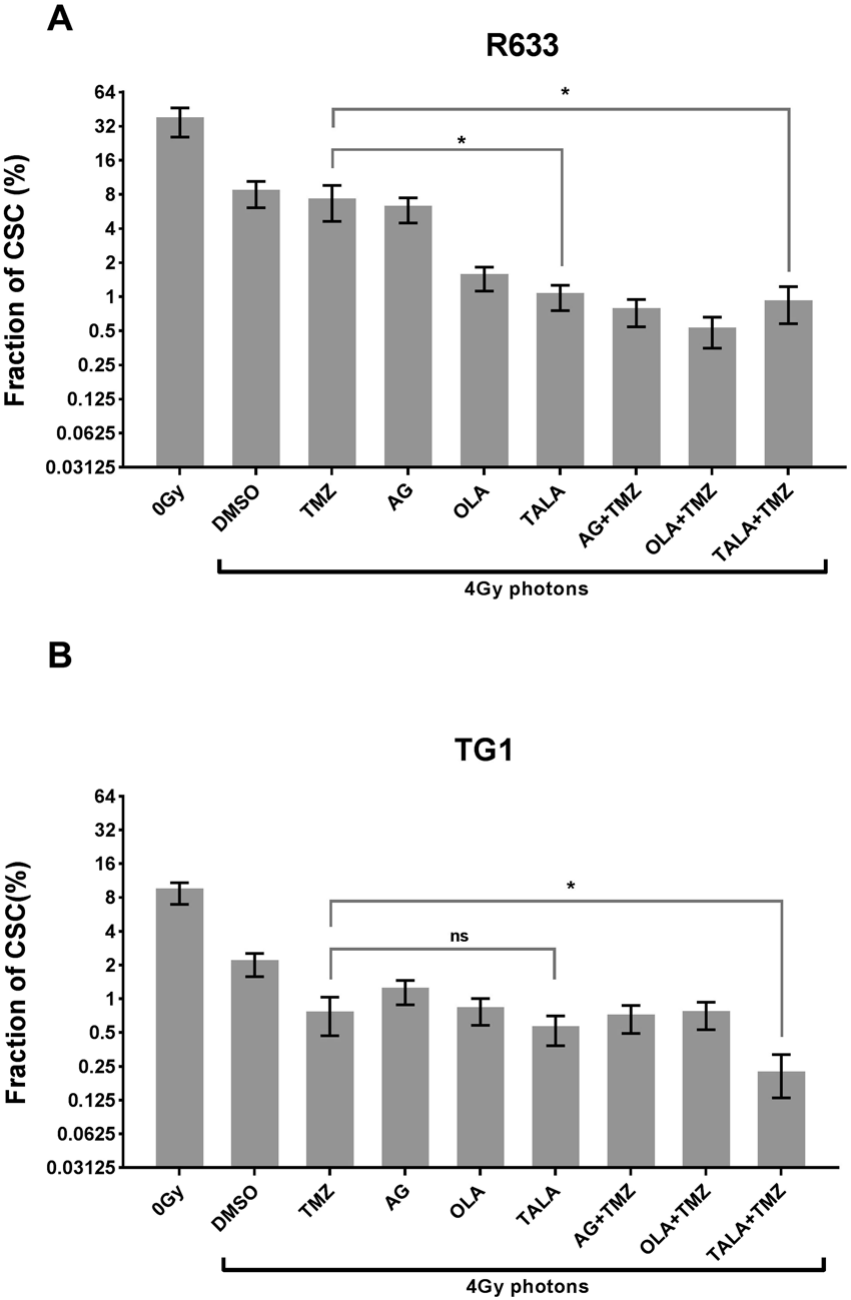
Impact of 4 Gy photonic irradiation in combination with talazoparib (TALA), temozolomide (TMZ), olaparib (OLA), or AG14361 (AG) on GSC fractions for the (**A**) R633 and (**B**) TG1 cell lines. Combinations with significant decrease (*p* < 0.05) in comparison with temozolomide +4 Gy are marked with *. Nonsignificant differences are marked with ns.

The estimated percentage of GSCs for R633 was 34% (Fig. 3A: Panel 0 Gy). To our knowledge, data on the R633 cell line have never been reported. For the TG1 cell line, 8.69% of cells were able to form neurospheres (Fig. 3B, Panel 0 Gy), consistent with previous reports^17^. That the 2 cell lines had different GSC fractions reflects inherent intertumoral heterogeneity and may explain the discrepancies in our study.

### Combinations of PARPi plus photonic irradiation decrease GSC frequency

Two hours after the beginning of exposure to the different radiosensitizers, cells were irradiated with 4 Gy photonic irradiation. For the R633 cell line (Fig. 3A), when compared with nonirradiated controls, 4 Gy photonic irradiation led to a decrease of about 80% in GSC percentage (34.6% *vs*. 7.9%, *p* < 0.001). When R temozolomide was used in combination with irradiation, the percentage of GSCs was not significantly different: 6.6% *vs* 7.9% for irradiation alone (*p* = 0.427). Combining irradiation with talazoparib alone or with talazoparib and temozolomide significantly decreased the GSC frequency, to 0.9% (*p* < 0.001) and 0.8% (*p* < 0.001), respectively. These last combinations were more efficient than the Stupp combination in reducing GSC fraction (*p* < 0.001) and were able to eliminate up to 97% of GSCs.

Talazoparib was compared with 2 other PARPis: olaparib and AG14361. For the R633 cell line, except in the case of AG 14361 delivered alone with irradiation (*p* = 0.07), all treatments significantly reduced GSC frequency in comparison with temozolomide + irradiation (Fig. 3A). A ranking of PARPi and PARPi plus temozolomide treatments can be proposed as follows, from the most radiosensitizing to the least:

- talazoparib > olaparib > AG14361
- olaparib + temozolomide > talazoparib + temozolomide = AG14361 + temozolomide
  For TG1 cells, a reduction in GSCs of about 80% was also observed after single irradiation, compared with the 0 Gy control sample (8.6% *vs*. 2%, *p* < 0.001) (Fig. 3B). Temozolomide combined with irradiation slightly but significantly decreased the GSC fraction from 2% to 0.7% (*p* < 0.0001). The combination of talazoparib with irradiation did not decrease GSC frequency in comparison with temozolomide + 4 Gy: 0.5% *vs*. 0.7% (*p* = 0.28). However, the triple combination of irradiation, talazoparib, and temozolomide was more efficient than the Stupp combination, with a reduction in GSC density to 0.2% (*p* < 0.001).
  For the TG1 cell line, none of the combinations with AG14361 and olaparib decreased the GSC subpopulation more than temozolomide plus irradiation (Fig. 3B). As with the R633 cell line, a ranking of PARPis can be established:
- talazoparib > olaparib > AG14361
- talazoparib + temozolomide > olaparib + temozolomide = AG14361 + temozolomide

Depending on the cell line, GSCs had different sensitivities to PARPis combined with radiotherapy. Whereas the benefit from PARPi combined with radiotherapy is very clear for the R633 cell line when compared with the Stupp combination, for the TG1 cell line, only 2 combination (talazoparib + temozolomide + 4 Gy) reduced GSCs more extensively than the Stupp combination.

### Carbon ion irradiation combined with talazoparib is highly efficient at decreasing GSC frequency

**Figure 4:**
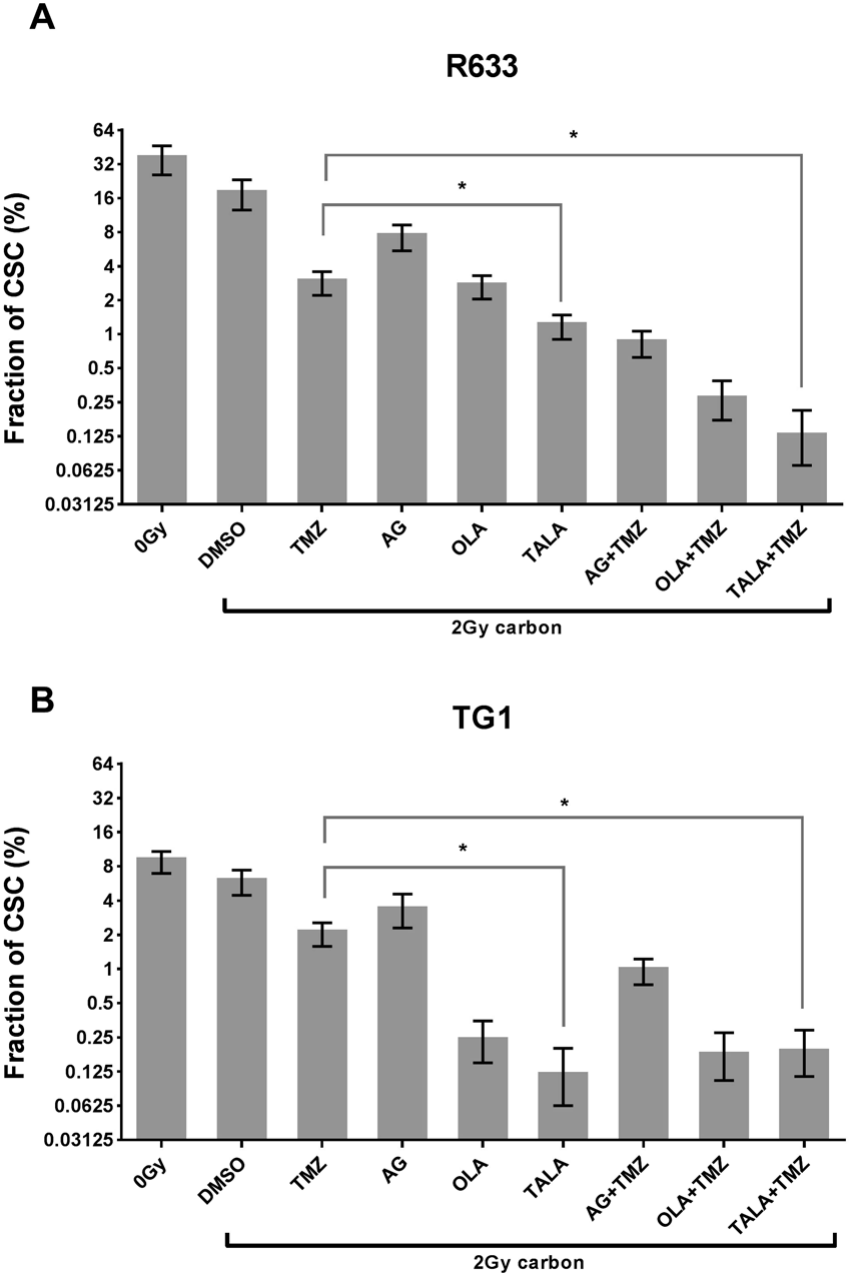
Impact of 2 Gy carbon irradiation in combination with talazoparib (TALA), temozolomide (TMZ), olaparib (OLA), or AG14361 (AG) on GSC fractions for the (**A**) R633 and (**B**) TG1 cell lines. Combinations with significant decrease (*p* < 0.05) in comparison with temozolomide + 4 Gy are marked with *. Nonsignificant differences are marked with ns.

To evaluate the impact of carbon ion irradiation in combination with PARPi, a single dose of 2 Gy was used, assuming as RBE ratio of around 2 with respect to photonic irradiation^14^. For the R633 cell line, 2 Gy carbon irradiation resulted in a GSC frequency of 17% (Fig. 4A). The addition of temozolomide to 2 Gy carbon irradiation significantly reduced the GSC fraction to 2.8% (p < 0,001).

Combinations of talazoparib and talazoparib + temozolomide with 2 Gy carbon irradiation were highly effective and led to residual GSC fractions of 1.2% and 0.1%, respectively. None of the other PARPis combined with carbon irradiation achieved greater GSC reduction than temozolomide + talazoparib + 2 Gy carbon (Fig. 4A). With or without temozolomide, talazoparib plus carbon irradiation systematically achieved a greater decrease in GSC fractions than olaparib and AG14361. A ranking from the most to least efficient PARPi or PARPi in combination with temozolomide can be proposed:

- talazoparib > olaparib > AG14361
- talazoparib + temozolomide > olaparib + temozolomide > AG14361 + temozolomide
  For the TG1 cell line, the GSC frequency after 2 Gy carbon irradiation alone was 5.7% (Fig. 4B). The combination of temozolomide with carbon irradiation decreased the GSC fraction further by 2.9-fold (2% vs. 5.7%, *p* < 0.001). When talazoparib was combined with carbon irradiation or with temozolomide + carbon irradiation, the GSC fraction was markedly reduced by 98% (*p* < 0.001) and 97% (*p* < 0.001), respectively, in comparison with 2 Gy carbon irradiation alone (Fig. 4B). As in the preceding photonic irradiation schedule, AG14361 and olaparib were also tested. Among all tested combinations, talazoparib with 2 Gy carbon irradiation elicited the greatest reduction in GSC frequency. Thus, we can consider that for the TG1 cell line:
- talazoparib > olaparib > AG14361
- talazoparib + temozolomide = olaparib + temozolomide > AG14361 + temozolomide

### Particle irradiation combined with PARPi decreases the GSC fraction further compared with photonic irradiation

**Figure 5:**
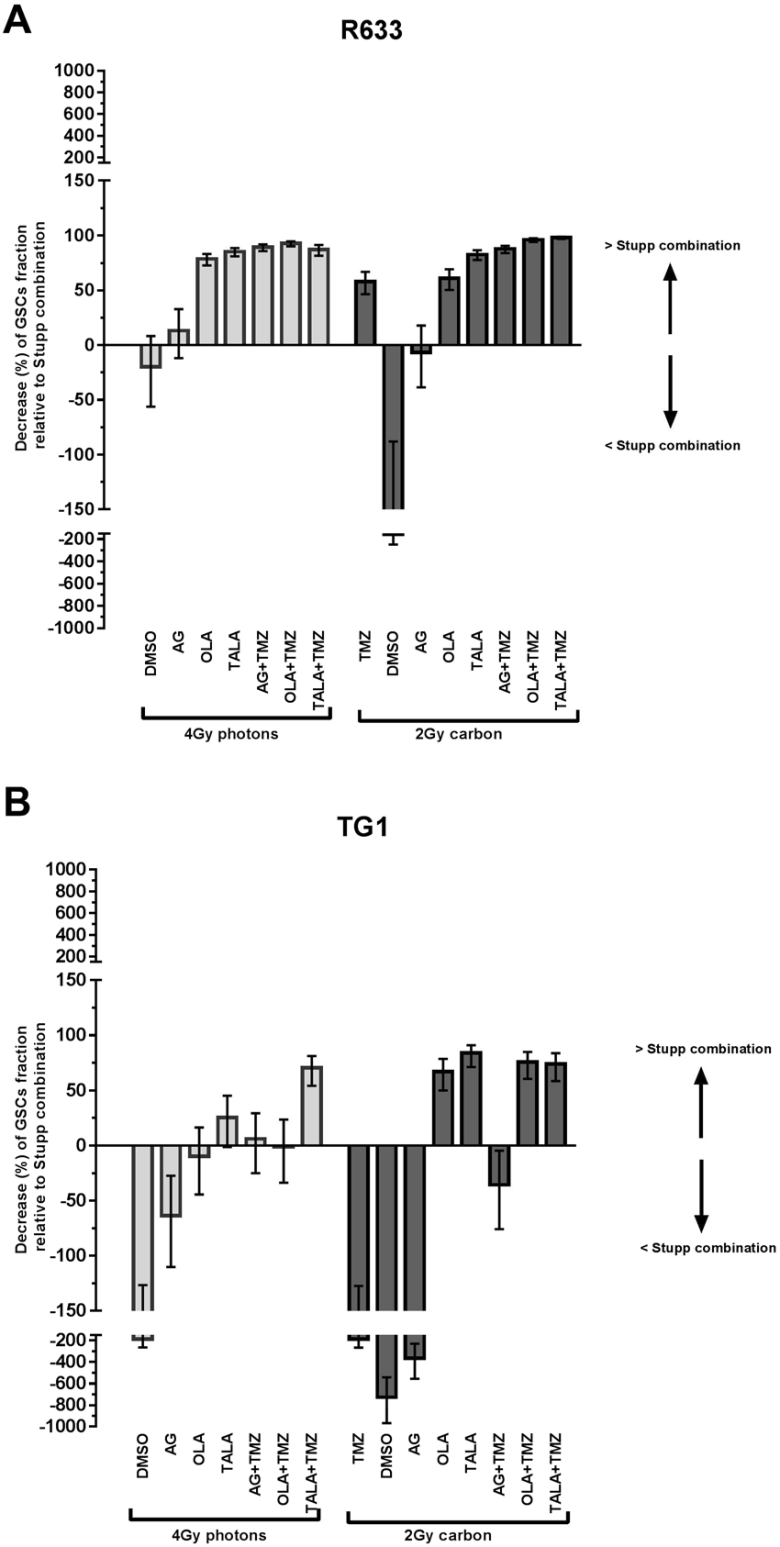
Impacts of all combinations on GSC reduction, normalized to Stupp combination. Positive values (histogram bar upwards) signify a greater GSC reduction than with the Stupp combination. (**A**) R633 cell line; (**B**) TG1 cell line.

For the R633 cell line, 2 Gy carbon and 4 Gy photonic irradiation led to GSC frequencies of 17% and 7.9% (*p* < 0.001), respectively (Figs 3A and 4A). Without any radiosensitizer, 2 Gy carbon irradiation had a significantly lower impact on GSC frequency than 4 Gy photonic irradiation. However, 2 Gy carbon irradiation combined with temozolomide reduced the GSC fraction from 6.6% to 2.8% (p < 0,001)in comparison with the Stupp combination. When talazoparib was combined with carbon irradiation or with temozolomide + carbon irradiation, the GSC fraction was reduced by 83%(p < 0,001) and 98%(p < 0,001), respectively, in comparison with the Stupp combination (Fig. 5A). Talazoparib plus irradiation had similar impacts on GSC frequency, regardless of the modality of irradiation: a reduction of between 83% and 85% was observed (*p* = 0.371). In contrast, when temozolomide was added to talazoparib, carbon irradiation was more efficient than photonic irradiation (reduction of 98% *vs*. 87%, *p* < 0.001). Considering all PARPis and all modalities of irradiation compared with the Stupp combination, the most notable decrease in GSC proportion was observed for carbon irradiation combined with talazoparib and temozolomide. Furthermore, except for AG14361 alone combined with irradiation, all combinations were significantly more efficient than the Stupp combination.

For the TG1 cell line, the GSC frequencies after 2 Gy carbon and 4 Gy photon irradiation were 5.7% and 1.9% (*p* < 0.001), respectively (Fig. 4B). As with the R633 cell line, 2 Gy carbon irradiation had a significantly lower impact on GSC frequency than 4 Gy photonic irradiation in the absence of any radiosensitizer.

The combination of temozolomide with carbon irradiation did not decrease the GSC fraction further when compared with the photonic Stupp combination (2% *vs*. 0.7%, *p* < 0.001). When talazoparib was combined with carbon irradiation or with temozolomide + carbon irradiation, the GSC fraction was reduced by 83% or 74%, respectively, in comparison with the Stupp combination (Fig. 5B). With photonic irradiation, talazoparib plus temozolomide was the only combination that was more efficient than the Stupp combination. With carbon irradiation, more combinations were effective in reducing GSC frequency. In fact, with or without temozolomide, talazoparib and olaparib decreased the GSC fraction by more than 65% with respect to the Stupp combination. The largest reduction in GSC proportion was observed for carbon irradiation combined with talazoparib (83%).

When 2 Gy carbon combinations were compared with temozolomide + 4 Gy photonic irradiation, which could be considered the standard schedule, the results were promising (Fig. 5A,B); regardless of cell line, most combinations of PARPis, particularly those that included talazoparib and olaparib, led to greater decrease in residual GSC fraction versus the Stupp combination.

## Discussion

In the present study, we have demonstrated that PARP inhibition with talazoparib is an effective method for radiosensitizing GBM cells and, particularly, the population of stem cells, which are renowned for their radioresistance and are at the origin of most recurrences^39^. Two modalities of irradiation were tested: classical photonic and innovative carbon ion irradiation. To our knowledge, there is no other published study that has evaluated talazoparib as a radiosensitizer; few phase I and *in vivo*/*in vitro* studies have examined talazoparib in combination with chemotherapy (carboplatin or temozolomide alone)^27,40,41^.

According to our results, talazoparib appears to be a better radiosensitizer than either AG14361 or olaparib, which are used at a much higher concentration. Indeed, regardless of the cell line and irradiation modality, talazoparib always reduced the GSC fraction more extensively than any other PARPi treatment. This higher efficacy could be explained by the specific ability to stay bound to the PARP1-DNA complex, increasing the DSB rate while the replication fork progresses^42^. However, it is now largely accepted that radio-induced cell death is linked to the rate of unrepaired DNA DSBs. Indeed, it was already shown that decreased viability of GSCs exposed to PARPi and irradiation may be explained by an increase of unrepaired double strand breaks. Venere and colleagues^43^ evaluated repair of DNA damage induced by irradiation in GSCs. GSCs pretreated with olaparib or left untreated, were irradiated, and the level of DNA damage was monitored by scoring γ-H2AX foci. Irradiated GSCs resolved damaged DNA by 24 h after IR as evaluated by the loss in γH2AX signal, whereas in GCS exposed to olaparib and irradiation, DNA damage repair was delayed. DNA repair kinetics were compromised and it led to continual formation of DNA breaks, as unrepaired ROS-induced damage which can be converted to double-strand breaks. Ahmed *et al*.^5^ also demonstrated that PARPi significantly impaired repair of radiation induced DSBs in GSC enriched cell population as demonstrated by a significant increase in the number of unresolved γ-H2AX foci 24 hours post-irradiation.

Furthermore, talazoparib possesses greater anti-PARP3 activity than olaparib or AG14361^42^, which could also explain in part its higher efficacy. Moreover, PARP3 inhibition alters signaling^44^ and DSB repair and promotes mutagenic alternative nonhomologous end joining instead of homologous recombination^45^. Recently, Day *et al*. proposed a model in which PARP3 suppresses G quadruplex (G4) DNA in response to DNA damage^46^. So, if PARP3 is inhibited, an accumulation of G4 DNA could occur, making accurate DNA repair via homologous recombination more difficult. As a consequence, the inhibition of PARP3 could confer hypersensitivity to radiotherapy, generating numerous DSBs; this could also contribute to explaining why talazoparib is more effective than the other PARPis tested.

For the 2 cell lines that were studied, a single irradiation of 2 Gy carbon was less effective than 4 Gy photonic irradiation in decreasing GSC fractions; the treatments were expected to have similar results, given the hypothesis of an RBE of approximately 2. However, when PARPi was added to carbon irradiation, the effect on GSC reduction was at least equivalent to the comparable photonic treatment (R633 cell line) or even superior (TG1 cell line). Thus, the radiosensitizing effect of PARPis, such as talazoparib, seems to be more pronounced with high LET irradiation than with classical irradiation, even if lower irradiation doses are used. For example, the decrease in the TG1 GSC fraction was 2.8- or 3.5-fold greater when talazoparib or olaparib was combined with 2 Gy carbon than with 4 Gy photon irradiation. These results are in accordance with the study from Hirai *et al*. on prostate cancer cells^47^. Indeed, these authors observed a 66% increase in the enhancement ratio when olaparib was combined with 70 KeV μm^−1^ LET carbon therapy instead of 13 KeV μm^−1^ low LET carbon therapy. Aside from DSBs, high LET irradiations induce more complex DNA damage than photons, called oxidative clustered DNA lesions (OCDLs). OCDLs include oxidized bases, apurinic-apyrimidinic sites, and SSBs, and these lesions are repaired mainly by BER, in which PARP plays a significant role^48^. In the presence of olaparib or talazoparib, this sublethal damage is not repaired and can lead to lethal damage. This could be the reason why the radiosensitizing effect of PARPi is more pronounced with charged particle irradiation.

These promising results give clinicians cause to hope for efficient strategies to treat glioblastoma and to increase the therapeutic ratio. Indeed, selected activity of PARPi on proliferating cells^11^ combined to balistic proprieties and higher Relative biological effect of Carbon ion beam, could improve treatment by reducing toxicity, particularly neurocognitive toxicity, while increasing the biological dose delivered to the clinical target volume. Given the actual low accessibility to clinical carbon beam, we have opened recently a phase I-IIa trial to investigate the toxicity and efficacy of PARPi and TMZ concomitantly with photonic radiotherapy in first line treatment of patients with unresectable high risk HGG (NCT03212742). A whole exome analysis of patients included is planned in this study. Correlation between treatment response and tumor genetic profiling will allow us to identify biomarkers that can be useful in treatment improvement and/or present a prognostic value. In 2022, we will be able to propose to our patients carbon beam irradiation, and definitely clinical carbon beam irradiation combined with PARPi will be the next clinical research step. However pending the arrival of this hadrontherapy facilities^49^ hadronbiology still needs exhaustive research. While it could be considered an efficient antitumor therapy^14^, the high sensitivity of human neural stem cells to carbon ion beams could limit its use in clinical practice^50^. The 2 cell lines analyzed in the present study have shown the same sensitivity to irradiation but very different responses to the radiosensitizing effect of PARPis. *PTEN*-deleted R633 cells were significantly more sensitive to PARPis, and *PTEN* deletion could be considered to be predictive of sensitivity. Similar observations have been made by other groups^51^. It is well known that *PTEN* has numerous nuclear functions, including transcriptional regulation of RAD51 recombinase, the product of which is essential for homologous recombination repair of DNA breaks. Thus, for *PTEN*-deleted tumors, PARPi combined with irradiation could lead to synthetic lethality and consequently to cell death. Accumulation of unrepaired DNA DSBs in the R633 cell line resulted in major G2/M arrest (Fig. 1). This enhanced G2/M block for the R633 cell line could even be increased by *P53* mutation, which impairs efficient G1 arrest. Thus, the G1 checkpoint is altered, and the cell cycle continues until the G2 checkpoint, leading to greater G2/M blockade^52^. Interestingly, this G2/M block persisted until at least 96 hours after irradiation, reflecting greater difficulty with DNA repair. These cells are blocked in the most radiosensitive cell phase, and for clinical practice, using a fractionated protocol could improve the efficacy of radiotherapy.. BRCA1-BRIP1 interaction is essential for limiting DNA damage tolerance. BRIP1 may serve to link FANCD2 to BRCA1, and thus to allow double strand break repair. The BRIP1 splicing site mutation found here, potentially could lead to an abnormal protein, and then also explain R633 cell line sensitivity to PARPi by promoting synthetic lethality.

Other biomarkers have been recently proposed such as IDH1-2 mutations. IDH mutations, found in about 3% of primary glioblastoma^53^, and for 80% of low grade glioma, were predictive of PARP inhibitor sensitivity in a range of clinically relevant models, including primary patient-derived glioma cells^54,55^.

In conclusion, we have shown that PARPis—in particular, talazoparib—may be considered new radiosensitizers for radioresistant GSCs, and their combination with high LET particle therapy is very promising. The use of PARPis combined with carbon beam irradiation drastically reduced the GSC frequency of GBM cell lines *in vitro*. Almost all PARPis combined with irradiation led to a lower GSC frequency when compared with the Stupp combination. In the context of *in vivo* models, a reduction in the GSC fraction could delay GBM recurrence or tumor growth. However, the delivery of talazoparib across the cerebral blood barrier could be an impediment in *in vivo* studies^41^. If our results are confirmed in *in vivo* studies, and if results ofphase I studies with olaparib and photonic radiotherapy keep its promises, then a phase I study could be designed with intensification strategies that combine particle therapy and talazoparib.

While the results described in this paper come from proof-of-principle experiments in an *in vitro* model, it will be important to extend these results to *in vivo* models to verify whether our conclusions are valid in a more “tumor-like” setting. To move in such direction, our experiences need to be repeated in 3D GBM models that include surrounding healthy tissue and *in vivo* models to assess toxicity in normal stem cells. We are thereby developing a bioprint model of GBM embedded in healthy tissues to investigate both toxicity of combined treatments in healthy tissues and anti tumoral efficiency in tumoral tissue. Moreover, a Crispr/Cas9 strategy will be used in order to define cell pathways involved in carbon ion and/or PARPi resistance.

## Electronic supplementary material

Supplementary table and figures

Supplementary dataset 1
