# Supplementary material for: Radiosensitization Effect of Talazoparib, a Parp Inhibitor, on Glioblastoma Stem Cells Exposed to Low and High Linear Energy Transfer Radiation
Source: Sci Rep. 2018 Feb 26;8:3664. doi: 10.1038/s41598-018-22022-4 (PMC5826933; doi:10.1038/s41598-018-22022-4)
Supplement: Supplementary file 1 — Supplementary table and figures [file 41598_2018_22022_MOESM1_ESM.docx]

**RADIOSENSITIZATION EFFECT OF TALAZOPARIB, A PARP INHIBITOR, ON GLIOBLASTOMA STEM CELLS EXPOSED TO LOW AND HIGH LINEAR ENERGY TRANSFER RADIATION**

Paul LESUEUR^1, 2^, François CHEVALIER^1^, Elias A.EL-HABR^3^, Marie-Pierre JUNIER^3^, Hervé CHNEIWEISS^3^, Laurent CASTERA^4,^ Etienne MULLER^4^, Dinu STEFAN^2^, and Yannick SAINTIGNY^1^

^1^ LARIA, CIMAP, CEA, Caen, France

^2^ Centre François Baclesse, Radiotherapy Unit, Caen, France

^3^ CNRS UMR8246, Inserm U1130, UPMC, Neuroscience Seine-IBPS, Sorbonne Universities, 75005 Paris, France

^4^ Centre François Baclesse, Plateforme de séquençage haut débit, Caen, France

| List of the 69 genes analyzed | | |
| --- | --- | --- |
| ARID1A | FANCD2 | PTEN |
| ARID1B | FANCE | RAD17 |
| ATM | FANCF | RAD50 |
| ATR | FANCG | RAD51 |
| ATRX | FANCI | RAD51B |
| AURKA | FANCL | RAD51C |
| BAP1 | FANCM | RAD51D |
| BARD1 | GATA3 | RAD54L |
| BLM | H2AFX | RAP80 |
| BRCA1 | HDAC1 | RBBP8 |
| BRCA2 | HDAC2 | RNF168 |
| BRCC3 | HDAC3 | RPA1 |
| BRIP1 | MAD2L2 | SHFM1 |
| CDK12 | MDC1 | SLX4 |
| CHEK1 | MERIT40 | SMARCA2 |
| CHEK2 | MRE11A | SMARCA4 |
| EMSY | NBN | TDG |
| ERCC1 | PALB2 | TOPBP1 |
| ERCC2 | PARP1 | TP53BP1 |
| FAM175A | PIK3CA | XPA |
| FANCA | PIK3R1 | XRCC2 |
| FANCB | PIK3R2 | XRCC3 |
| FANCC | PPP2R2A | XRCC4 |

Supplementary Table S1: List of the 69 genes sequenced for exon analysis.


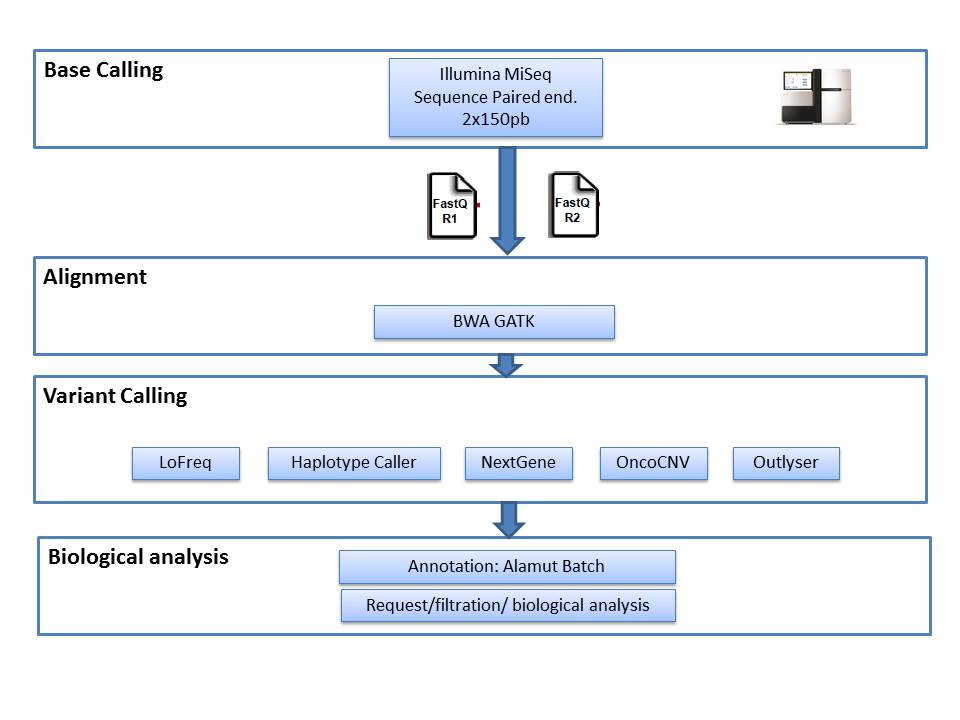


Supplementary figure S1: Pipeline used for exon sequencing analysis.


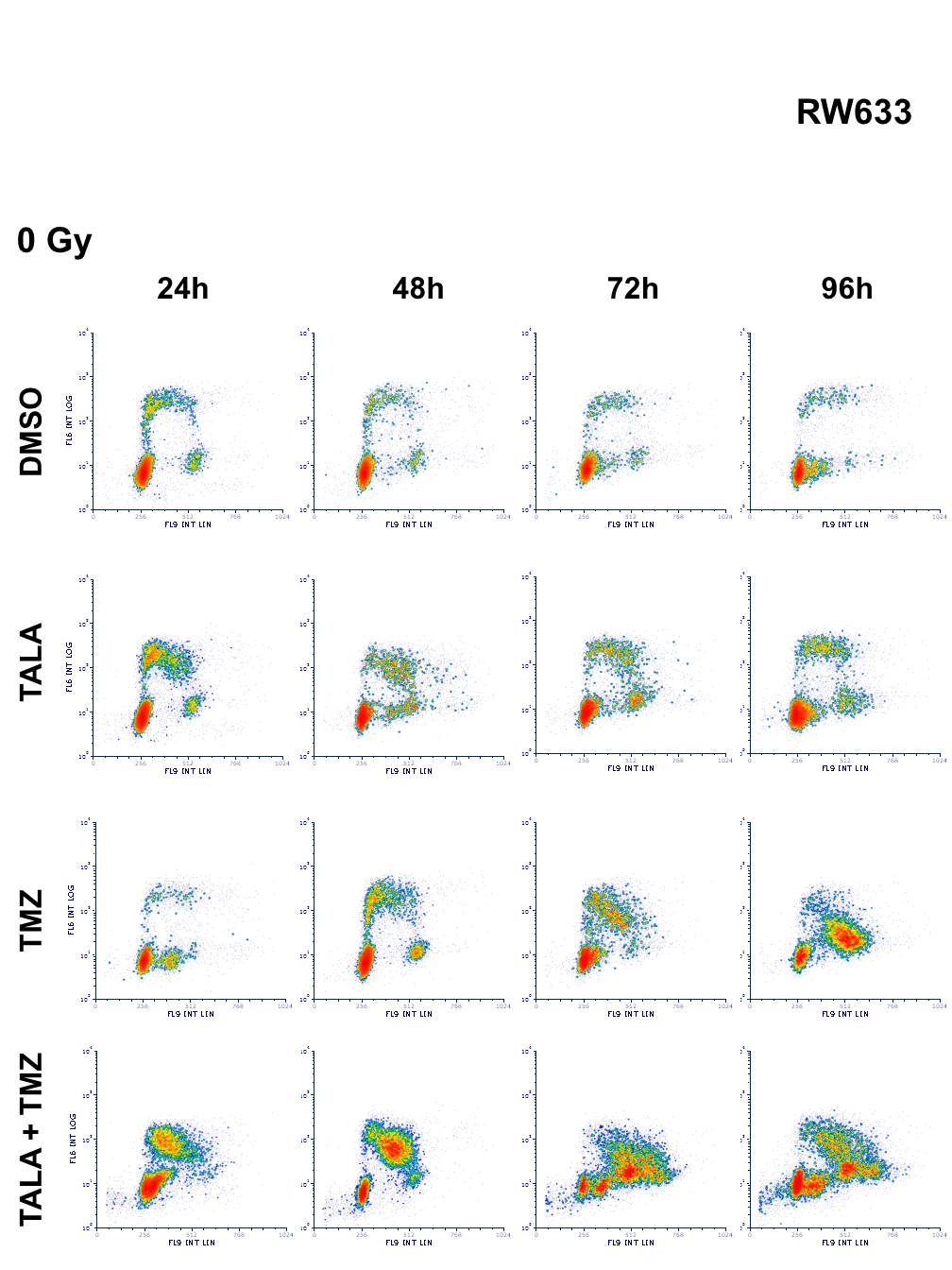


Supplementary figure S2: EDU/violet stain cell cycle analysis of R633 cells without irradiation.


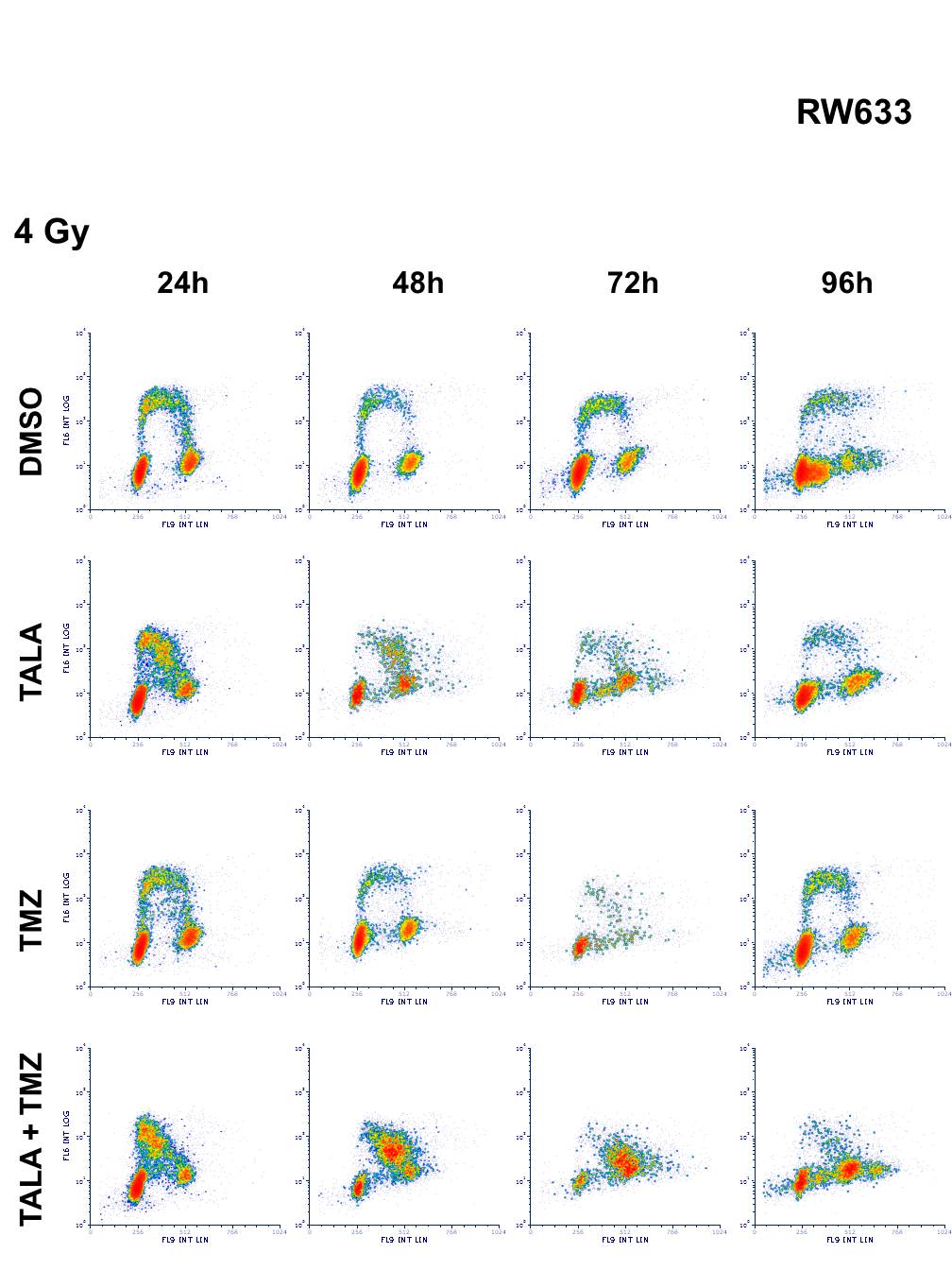


Supplementary figure S3: EDU/violet stain cell cycle analysis of R633 cells with 4 Gy photonic irradiation.


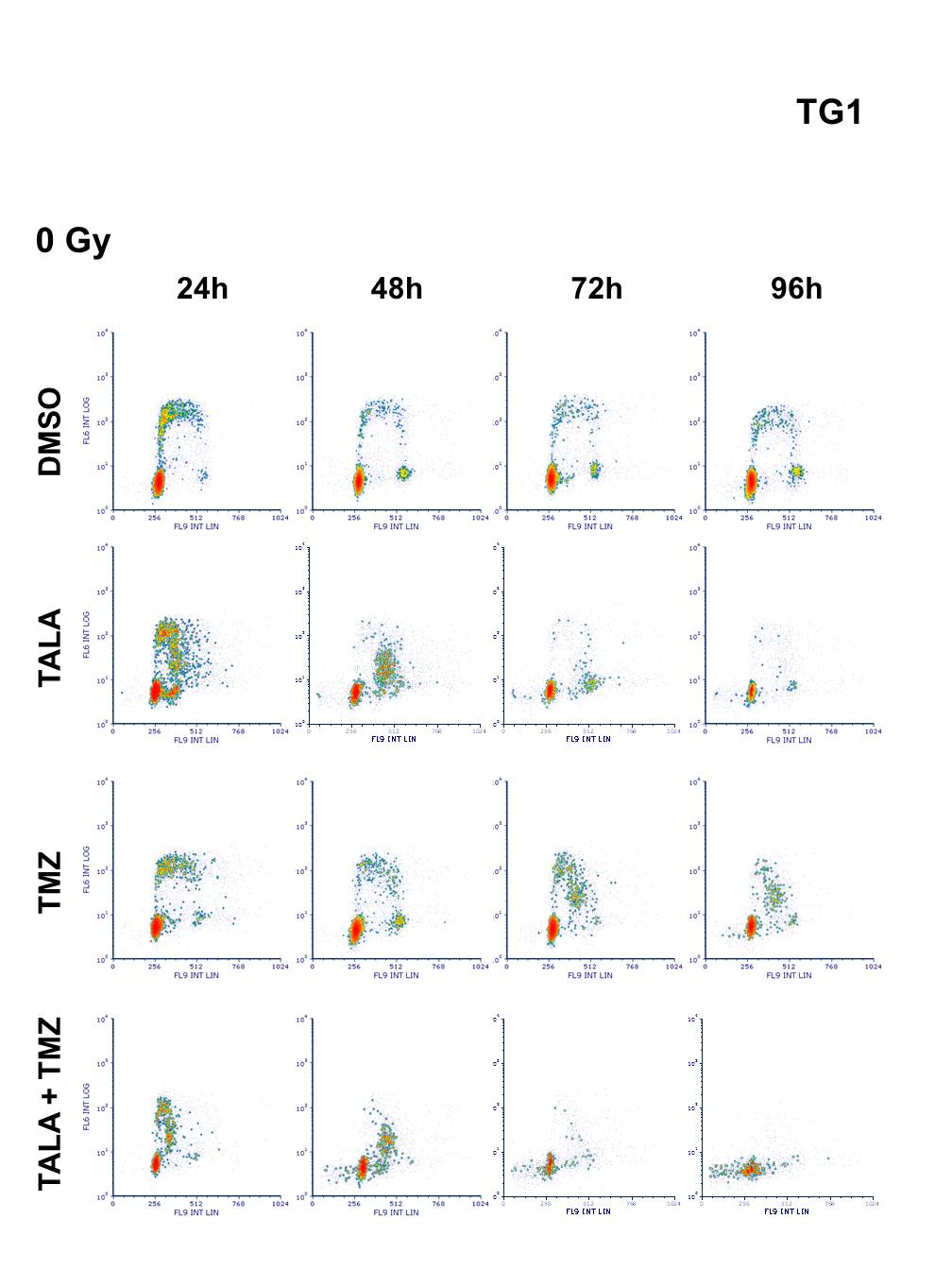


Supplementary figure S4: EDU/violet stain cell cycle analysis of TG1 cells without irradiation.


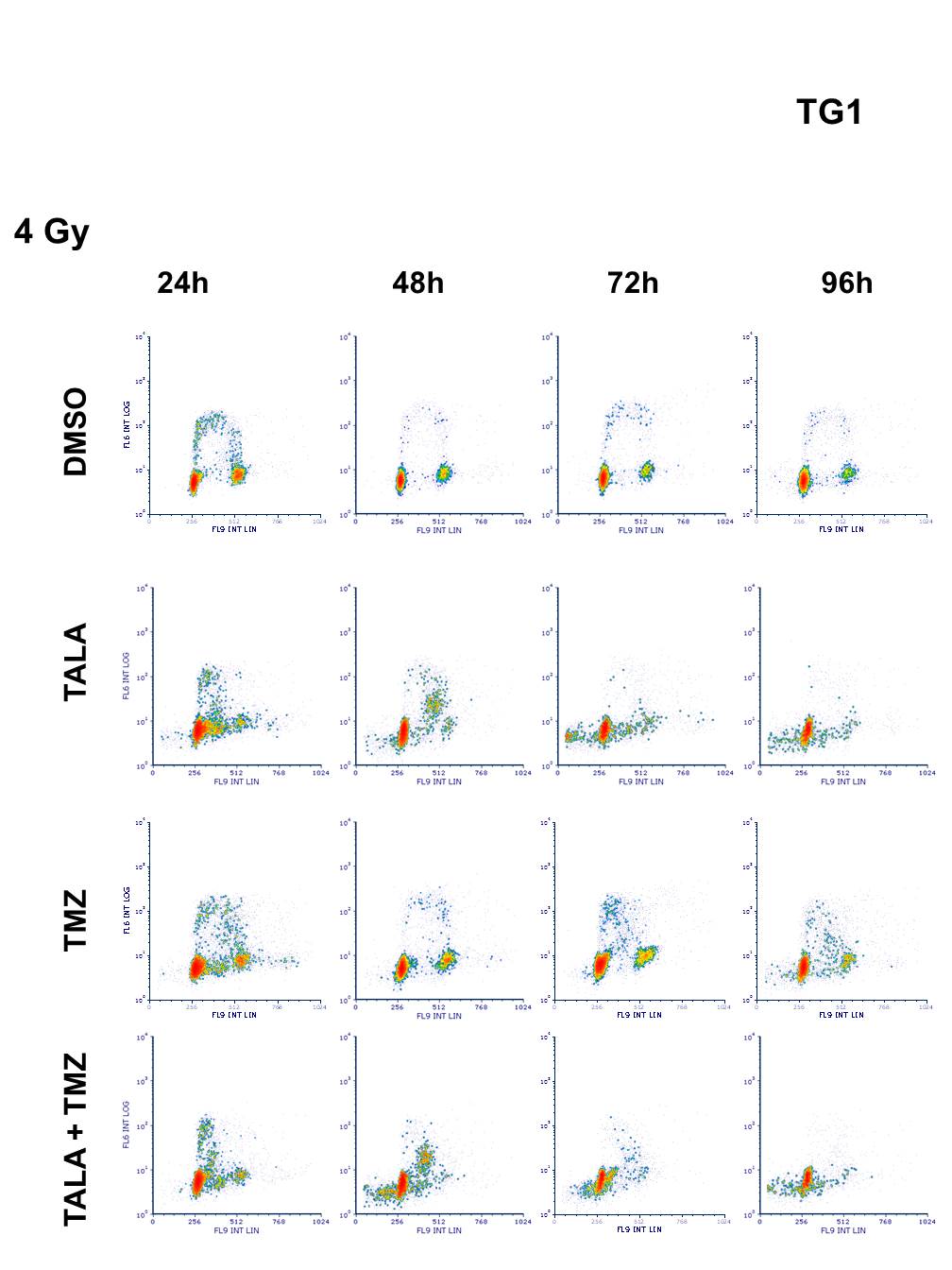


Supplementary figure S5: EDU/violet stain cell cycle analysis of TG1 cells with 4 Gy photonic irradiation.
